# Contemporary in-hospital and long-term prognosis of patients with acute ST-elevation myocardial infarction depending on renal function: a retrospective analysis

**DOI:** 10.1186/s12872-023-03084-3

**Published:** 2023-02-02

**Authors:** Christiane Engelbertz, Jannik Feld, Lena Makowski, Leonie Kühnemund, Alicia Jeanette Fischer, Stefan A. Lange, Christian Günster, Patrik Dröge, Thomas Ruhnke, Joachim Gerß, Eva Freisinger, Holger Reinecke, Jeanette Köppe

**Affiliations:** 1 Department of Cardiology I – Coronary and Peripheral Vascular Disease, Heart Failure, University Hospital Muenster, Cardiol, Albert-Schweitzer-Campus 1, Gebäude A1, 48149 Muenster, Germany; 2 Institute of Biostatistics and Clinical Research, University of Muenster, Muenster, Germany; 3 Department of Cardiology III - Adult Congenital and Valvular Heart Disease, University Hospital Muenster, Cardiol, Muenster, Germany; 4 AOK Research Institute, Berlin, Germany

**Keywords:** Acute myocardial infarction, Chronic kidney disease, 30-day mortality, Overall survival, Real world data

## Abstract

**Background:**

Cardiovascular disease is often associated with chronic kidney disease (CKD), resulting in an increased risk for poor outcome. We sought to determine short-term mortality and overall survival in ST-elevation myocardial infarction (STEMI) patients with different stages of CKD.

**Methods:**

In our retrospective cohort study with health insurance claims data of the Allgemeine Ortskrankenkasse (AOK), anonymized data of all STEMI patients hospitalized between 2010 and 2017 were analyzed regarding presence and severity of concomitant CKD.

**Results:**

A total of 175,187 patients had an index-hospitalisation for STEMI (without CKD: 78.6% patients, CKD stage 1: 0.8%, CKD stage 2: 4.8%, CKD stage 3: 11.7%, CKD stage 4: 2.8%, CKD stage 5: 0.7%, CKD stage 5d: 0.6%). Patients with CKD were older and had more co-morbidities than patients without CKD. With increasing CKD severity, patients received less revascularization therapies (91.2%, 85.9%, 87.0%, 81.8%, 71.7%, 76.9% and 78.6% respectively, *p* < 0.001). After 1 year, guideline-recommended medications were prescribed less frequently in advanced CKD (83.4%, 79.3%, 81.5%, 74.7%, 65.0%, 59.4% and 53.7%, respectively, *p* < 0.001). CKD stages 4, 5 and 5d as well as chronic limb threatening ischemia (CLTI) were associated with decreased overall survival [CKD stage 4: hazard ratio (HR) 1.72; 95% CI 1.66–1.78; CKD stage 5: HR 2.55; 95% CI 2.37–2.73; CKD stage 5d: 5.64; 95% CI 5.42–5.86; CLTI: 2.06; 95% CI 1.98–2.13; all *p* < 0.001].

**Conclusions:**

CKD is a frequent co-morbidity in patients with STEMI and is associated with a worse prognosis especially in advanced stages. Guideline-recommended therapies in patients with STEMI and CKD are still underused.

**Supplementary Information:**

The online version contains supplementary material available at 10.1186/s12872-023-03084-3.

## Background

Ischemic heart disease is a major cause of cardiovascular mortality worldwide [1]. In Germany, the incidence of ST-segment elevation myocardial infarction (STEMI) decreased during the last decades while the incidence of non-ST-segment elevation myocardial infarction increased in the same time period [2]. Although guideline-recommended treatment with percutaneous coronary intervention (PCI) and drugs like beta-blockers, angiotensin-converting-enzyme (ACE) inhibitors or angiotensin II receptor blockers (ARB) as well as statins and antiplatelet drugs [3, 4] were applied more frequently [5], in-hospital mortality rates of STEMI patients remained rather stable [2, 5].

Cardiovascular disease is frequently associated with chronic kidney disease (CKD) due to the same risk factors, e.g. older age, arterial hypertension, diabetes mellitus or dyslipidemia, and vice versa [6, 7]. Patients with STEMI and CKD are at increased risk for poor outcome: they often suffer from a high complexity of concomitant diseases and they are frequently undertreated, because they are often excluded from randomized controlled trials resulting in a poor evidence base for specialized treatment. Furthermore, patients suffering from CKD are less likely to undergo PCI due to the fear of acute kidney injury after contrast media use [6–8], although guidelines recommend a PCI as the first choice of reperfusion therapy, even in patients with kidney disease [2].

The real-world treatment and prognosis of STEMI patients have been evaluated by numerous registries [5, 8–12]. While these registries analyzed treatment strategies, in-hospital mortality and 1-year mortality of STEMI patients also with focus on renal insufficiency, they lack a detailed analysis of patients according to their CKD stages and an analysis of the long-term outcome of STEMI patients with CKD. CKD is a progressive disease with a wide range of severity, starting with CKD stage 1 with kidney damage but no impaired estimated glomerular filtration rate (eGFR) and ending with CKD stage 5 with dialysis-depended kidney failure. Hence, a detailed analysis of these patients’ characteristics, the usage of guideline-recommended reperfusion and drug therapies and outcome after STEMI is needed to improve understanding and treatment. Therefore, we analyzed claims data of all STEMI patients with in-hospital admission between 2010 and 2017 according to their CKD stage and with focus on application of guideline recommended therapies as well as 30-day mortality and overall survival.

## Methods

The German reimbursement of inpatient hospital services is based on the “German Diagnosis Related Groups” (G-DRG) system using – among others – all in-hospital encoded diagnoses (classified by the International Statistical Classification of Diseases, German Modification, ICD-10 GM) and procedures (classified by the German procedure classification “Operationen- und Prozeduren-Schlüssel”, OPS). The ICD-10 GM codes for main and secondary diagnoses as well as the OPS codes for surgical and non-surgical procedures are recorded in detail by each hospital and thereafter provided to the health insurance. Outpatient information on diagnoses and procedures were also available in the dataset and prescribed medications were registered using the Anatomical Therapeutic Chemical (ATC) system. This specific and very precise documentation system allows the analysis of health insurance claims. For the present study, we analyzed anonymized data of the Federal Association of the Local Health Insurance Funds (Allgemeine Ortskrankenkasse – AOK). The AOK is a group of German health insurances and provides statutory health insurance for roughly 32% of the German population [13]. All applied ICD-10 GM, OPS, and ATC codes are listed in the Additional file 1: Table S1.

### Patient selection and baseline characteristics

All patients ≥ 18 years hospitalized with an encoded main diagnosis of STEMI (ICD-10 GM code I21.0, I21.1; I21.2, I22.0, I22.1, I22.8) in the years 2010 to 2017 were included in this retrospective analysis. The first hospitalization in this period was defined as the index-hospitalization. Patients were grouped according to their renal function defined as CKD stage 1 (ICD-10-GM code before 2010: N18.81, since 2010: N18.1), CKD stage 2 (before 2010: N18.82, since 2010 N18.2), CKD stage 3 (before 2010: N18.83, since 2010 N18.3), CKD stage 4 (before 2010: N18.84, since 2010 N18.4), CKD stage 5 (before 2010: N18.0, since 2010 N18.5; no OPS codes for dialysis), and no CKD (absence of any of these codes). Patients were classified to one of these 6 groups if no OPS-codes for dialysis were present. For patients on dialysis, we defined the group CKD stage 5d who presented with OPS-codes for dialysis at least twelve times within two years prior to index-hospitalization (OPS codes 8-853, 8–854, 8–855, 8–857) regardless of any code for renal insufficiency. If no ICD-code for renal insufficiency was recorded at index-hospitalization, we searched for any ICD-code for renal insufficiency within the two years prior index-hospitalization and used for classification that ICD-code which was timely closest to the index-hospitalization.

Baseline characteristics included numerous encoded diagnoses, e.g. for hypertension, diabetes mellitus, dyslipidemia, atrial fibrillation, at or within two years before index-hospitalization as well as procedures, i.e. percutaneous coronary intervention, within 2 years before index-hospitalization. Patients with inconsistent data covering this preliminary phase before the index-hospitalization were excluded. All codes are listed in the Additional file 1: Table S1.

### In-hospital treatment, outcome, medication and follow-up

All OPS-encoded procedures during the hospital stay as well as the diagnostic codes for shock, stroke, bleeding, sepsis, acute renal failure, and death, were captured to analyse in-hospital treatment and outcome. Medication was assessed on the basis of the ATC classification system. An ATC code within 90 days prior to index-hospitalization was regarded as a prescribed drug at index-hospitalization/admission. Patients were followed up from the date of admission of the first hospitalization with the main diagnosis STEMI until the end of follow-up (31 Dec 2018, exit from the data base, death), resulting in a median follow-up period of five years. If incomplete or inconsistent data were noticed during follow-up, patients were censored from this time point ongoing.

### Statistical analysis

As the primary questions of the study at hand, the association of the renal insufficiency stage on 30-day mortality and overall survival adjusted by patient’s risk profile after STEMI were analyzed. Therefore, a multivariable logistic regression model including age, sex, CKD stages and patient risk profile was performed to determine odd ratios (OR) for all CKD stages compared to patients without CKD. To address the association between CKD stage and overall survival, a multivariable Cox regression with time-dependent co-variables was performed to estimate hazard ratios (HR) for all CKD stages, also accounting for worsening of the patient's risk profile during follow-up. We also performed a multivariable Cox regression with time-dependent co-variables for the endpoint major cardiovascular events (MACE), defined as myocardial infarction, stroke, resuscitation and death.

Moreover, differences between categorical and continuous variable (e.g. co-morbidities at baseline or in-hospital outcome) between renal insufficiency stages were tested via Chi-square test and Kruskal–Wallis test, respectively. The observed survival rates were determined by a Kaplan Meier estimate. As a sensitivity analysis, the adjusted survival probability was additional determined by a multivariable Cox regression model including age, sex, CKD stage, and the patient's risk profile at the time of the index-hospitalization. The rate of prescribed medication one year after discharge was evaluated by determining the cumulative incidence function one year after discharge using Nelson-Aalen estimate. Death was thereby considered to be a competing risk event and differences between CKD stages were tested via Gray’s test.

All analyses were fully exploratory (hypotheses generating), not confirmatory, and an adjustment for multiple testing was not performed. Statistical analyses were performed using SAS software V9.4, SAS Institute Inc., Cary, NC, USA and R version 3.6.0, R foundation, Vienna, Austria.

## Results

### Baseline characteristics

From Jan 1^st^, 2010 to Dec 31^st^, 2017, a total of 175,187 patients were identified with an index-hospitalization for STEMI. Most of the patients had no encoded diagnosis of CKD (137,682 patients, 78.6%, Table 1), followed by 20,459 (11.7%) patients with CKD stage 3, 8347 (4.8%) patients with CKD stage 2 and 4960 (2.8%) patients with CKD stage 4. The CKD stages 1, 5, and 5d each comprised of less than 1500 patients (< 1.0%). An overview of the annual numbers of STEMI patients according to their renal status is given in the Additional file 1: Table S2.

**Table 1:** Baselines characteristics of patients with an index-hospitalization due to STEMI in 2010 to 2017

	No CKD	CKD Stage 1	CKD Stage 2	CKD Stage 3	CKD Stage 4	CKD Stage 5	CKD Stage 5D	*p* value
Patients, n (%)	137,682 (78.6)	1480 (0.8)	8347 (4.8)	20,458 (11.7)	4960 (2.8)	1295 (0.7)	965 (0.6)	
Female sex, n (%)	44,715 (32.5)	556 (37.6)	3258 (39.0)	9806 (47.9)	2767 (55.8)	511 (39.5)	334 (34.6)	< 0.001
Median age, yrs (IQR)	65 (21)	73 (18)	75 (16)	79 (12)	81 (11)	76 (14)	72 (18)	< 0.001
*Concomitant diseases recorded by ICD codes up to two years before STEMI*								
Coronary artery disease, n (%)	61,756 (44.9)	970 (65.5)	5139 (61.6)	13,013 (63.6)	3119 (62.9)	815 (62.9)	760 (78.8)	< 0.001
Hypertension, n (%)	114,014 (82.8)	1414 (95.5)	7,825 (93.7)	19,747 (96.5)	4834 (97.5)	1255 (96.9)	*	< 0.001
Diabetes mellitus, n (%)	45,691 (33.2)	926 (62.6)	4478 (53.6)	12,136 (59.3)	3208 (64.7)	856 (66.1)	628 (65.1)	< 0.001
Dyslipidemia, n (%)	98,882 (71.8)	1218 (82.3)	6514 (78.0)	15,745 (77.0)	3673 (74.1)	992 (76.6)	765 (79.3)	< 0.001
Obesity, n (%)	32,454 (23.6)	556 (37.6)	2820 (33.8)	6832 (33.4)	1690 (34.1)	485 (37.5)	333 (34.5)	< 0.001
Nicotine abuse, n (%)	37,826 (27.5)	325 (22.0)	1665 (19.9)	2777 (13.6)	563 (11.4)	207 (16.0)	203 (21.0)	< 0.001
Atrial fibrillation/flutter, n (%)	21,509 (15.6)	389 (26.3)	2365 (28.3)	7592 (37.1)	2082 (42.0)	543 (41.9)	437 (45.3)	< 0.001
Chronic heart failure, n (%)	56,644 (41.1)	918 (62.0)	5469 (65.5)	14,784 (72.3)	3959 (79.8)	1025 (79.2)	737 (76.4)	< 0.001
Right heart failure, n (%)	8428 (6.1)	197 (13.3)	1194 (14.3)	4503 (22.0)	1467 (29.6)	393 (30.3)	252 (26.1)	< 0.001
Left heart failure, n (%)								< 0.001
None	89,984 (65.4)	695 (47.0)	3572 (42.8)	7699 (37.6)	1532 (30.9)	*	322 (33.4)	
NYHA 1	4232 (3.1)	56 (3.8)	261 (3.1)	443 (2.2)	56 (1.1)	*	18 (1.9)	
NYHA 2	11,250 (8.2)	145 (9.8)	1077 (12.9)	1829 (8.9)	266 (5.8)	*	74 (7.7)	
NYHA 3	13,813 (10.0)	240 (16.2)	1458 (17.5)	3692 (18.0)	837 (16.9)	*	185 (19.2)	
NYHA 4	18,403 (13.4)	344 (23.2)	1979 (23.7)	6795 (33.2)	2249 (45.3)	*	366 (37.9)	
Lower extremity artery disease, n (%)								< 0.001
No LEAD	128,741 (93.5)	1299 (87.8)	7182 (86.0)	17,117 (83.7)	3993 (80.5)	965 (74.5)	597 (61.9)	
LEAD 1-3	8010 (5.8)	155 (10.5)	996 (11.9)	2791 (13.6)	789 (15.9)	257 (19.8)	263 (27.3)	
LEAD 4–6	931 (0.7)	26 (1.8)	169 (2.0)	550 (2.7)	178 (3.6)	73 (5.6)	105 (10.9)	
Cancer, n (%)	19,027 (13.8)	305 (20.6)	1807 (21.6)	5096 (24.9)	1220 (24.6)	324 (25.0)	249 (25.8)	< 0.001
*Previous events and interventions recorded by ICD codes and OPS-codes up to two years before STEMI*								
Previous cerebrovascular disease, n (%)	10,706 (7.8)	209 (14.1)	1201 (14.4)	3438 (16.8)	876 (17.7)	213 (16.4)	245 (25.4)	< 0.001
Previous stroke, n (%)	10,221 (7.4)	209 (14.1)	1231 (14.7)	3880 (19.0)	1025 (20.7)	227 (17.5)	237 (24.6)	< 0.001
Previous myocardial infarction, n (%)	11,152 (8.1)	200 (13.5)	1062 (12.7)	2811 (13.7)	687 (13.9)	191 (14.7)	240 (24.9)	< 0.001
Previous percutaneous coronary intervention, n (%)	3668 (2.7)	79 (5.3)	573 (6.9)	1380 (6.7)	265 (5.3)	104 (8.0)	189 (19.6)	< 0.001
Previous coronary artery bypass grafting, n (%)	3945 (2.9)	108 (7.3)	573 (6.9)	1647 (8.1)	389 (7.8)	144 (11.1)	149 (15.4)	< 0.001
Previous valve replacement, n (%)	464 (0.3)	11 (0.7)	91 (1.1)	281 (1.4)	61 (1.2)	14 (1.1)	42 (4.4)	< 0.001

CKD denotes chronic kidney disease; ICD, International Statistical Classification of Disease; LEAD, lower extremity artery disease; NYHA, New York Heart Association; OPS, Operationen- und Prozedurenschlüssel; STEMI, ST elevation myocardial infarction. Differences between CKD stages for categorical and continuous variables were tested via Chi-square test and Kruskal–Wallis test, respectively

*No results due to data safety reasons

Baseline characteristics of STEMI patients according to their renal status are shown in Table 1. Patients with a diagnosis code for CKD were older than patients without CKD (median age 72 (interquartile range [IQR] 18) to 81 (IQR 11) years for patients with CKD vs. 65 (IQR 21) years for patients without CKD, *p* < 0.001) and suffered from more cardiovascular comorbidities. Especially the prevalences of diabetes mellitus, atrial fibrillation and/or flutter, chronic heart failure, and lower extremity arterial disease were markedly increased in patients with CKD compared to patients without CKD. Previous stroke, previous myocardial infarction, and previous percutaneous coronary interventions were also documented more often in patients with all stages of renal insufficiency than in patient with normal renal function (all *p* < 0.001). The frequency of some of these concomitant diagnoses increased markedly with decreasing renal function, e.g. atrial fibrillation/flutter, chronic heart failure, lower extremity arterial disease, and previous stroke (all *p* < 0.001).

### Medication on admission

On admission, patients with CKD were treated with platelet inhibitors and/or oral anticoagulation, ACE inhibitors and/or ARB, beta-blockers, or statins at a higher rate than patients without CKD (all *p* < 0.001). In addition, the combination of all of these four guideline-recommended drug classes was prescribed more often to patients with CKD than to patients without CKD, increasing from 5.1% in patients with no CKD to 16.8% in patients with CKD stage 5d (*p* < 0.001; Additional file 1: Table S3).

### In-hospital treatment and complications

In-hospital treatment and complications differed between patients with CKD and patients without CKD (Table 2). Overall, patients with CKD received less reperfusion therapies although they presented more often with three-vessel disease (CKD stage 1: 42.4%, CKD stage 2: 41.6%, CKD stage 3: 43.8%, CKD stage 4: 42.7%, CKD stage 5: 45.3%, CKD stage 5d: 50.7%) than patients without CKD (32.7%; *p* < 0.001). Moreover, patients with CKD needed more medical emergency therapies and intensive care. They suffered more often from serious complications than patients without CKD. The lowest rates for guideline-recommended coronary angiography and percutaneous coronary intervention were reported in patients with CKD stage 4 (70.7% resp. 62.0%), whereas the highest rates were registered for patients without CKD (90.0% resp. 84.4%; both *p* < 0.001). Likewise, the frequency of stenting with drug-eluting stents was the lowest in patients with CKD stage 4 and the highest in patients without CKD (Table 2). Complications like sepsis, the combination of blood transfusion and/or bleeding as well as acute renal failure and/or renal replacement therapy increased with decreasing renal function (Table 2). Mean costs of in-hospital treatment was lowest in patients without CKD (8902 ± 14,789 €) and highest in patients with CKD stage 5 (25,029 ± 39,673 €). Interestingly, mean costs of dialysis-dependent patients were with 15,183 ± 26,186 € considerably lower than of patients with CKD stage 5.

**Table 2:** In-hospital treatment and outcome

Parameter	No CKD	CKD Stage 1	CKD Stage 2	CKD Stage 3	CKD Stage 4	CKD Stage 5	CKD Stage 5D	*p* value
Patients, n (%)	137,682 (78.6)	1480 (0.8)	8347 (4.8)	20,458 (11.7)	4960 (2.8)	1295 (0.7)	965 (0.6)	
Mean length of hospital stay, years (± SD)	10.9 (± 13.2)	12.4 (± 12.9)	13.3 (± 14.9)	15.2 (± 16.3)	17.1 (± 20.6)	25.1 (± 29.4)	16.9 (± 24.1)	< 0.001
Mean costs, Euro (± SD)	8902 (± 14,789)	9556 (± 12,081)	10,050 (± 15,794)	11,126 (± 17,867)	13,323 (± 25,111)	25,029 (± 39,673)	15,183 (± 26,186)	< 0.001
Coronary artery disease								< 0.001
One-vessel disease, n (%)	39,760 (28.9)	292 (19.7)	1734 (20.8)	3284 (16.1)	689 (13.9)	167 (12.9)	135 (14.0)	
Two-vessel disease, n (%)	36,125 (26.2)	353 (23.9)	1997 (23.9)	4511 (22.1)	841 (17.0)	247 (19.1)	164 (17.0)	
Three-vessel disease, n (%)	45,005 (32.7)	628 (42.4)	3476 (41.6)	8952 (43.8)	2118 (42.7)	587 (45.3)	489 (50.7)	
Unknown, n (%)	16,792 (12.2)	207 (14.0)	1140 (13.7)	3711 (18.1)	1312 (26.5)	294 (22.7)	177 (18.3)	
Coronary angiography, n (%)	123,858 (90.0)	1261 (85.2)	7171 (85.9)	16,530 (80.8)	3508 (70.7)	982 (75.8)	741 (76.8)	< 0.001
Percutaneous coronary intervention, n (%)	116,204 (84.4)	1138 (76.9)	6510 (78.0)	14,886 (72.8)	3076 (62.0)	834 (64.4)	632 (65.5)	< 0.001
Drug-eluting stent, n (%)	83,064 (71.5)	792 (69.6)	4458 (68.5)	9680 (65.0)	1860 (60.5)	519 (62.2)	407 (64.4)	< 0.001
Bare-metal stent only, n (%)	26,160 (22.5)	256 (22.5)	1560 (24.0)	3963 (26.6)	914 (29.7)	230 (27.6)	142 (22.5)	0.004
Coronary artery bypass grafting, n (%)	6164 (4.5)	111 (7.5)	512 (6.1)	1137 (5.6)	265 (5.3)	124 (9.6)	75 (7.8)	< 0.001
Intervention and/or thrombolysis, n (%)	125,539 (91.2))	1271 (85.9)	7260 (87.0)	16,725 (81.8)	3557 (71.7)	996 (76.9)	758 (78.6)	< 0.001
Intra-aortic balloon pump, n (%)	3847 (2.8)	59 (4.0)	295 (3.5)	810 (4.0)	246 (5.0)	112 (8.7)	42 (4.4)	< 0.001
Extracorporeal membrane oxygenation, n (%)	1280 (0.9)	13 (0.9)	66 (0.8)	213 (1.0)	76 (1.5)	37 (2.9)	18 (1.9)	< 0.001
Cardiogenic shock, n (%)	17,224 (12.5)	230 (15.5)	1175 (14.1)	3597 (17.6)	1154 (23.3)	359 (27.7)	206 (21.4)	< 0.001
Impella and/or intra-aortic balloon pump and/or extracorporeal membrane oxygenation or shock, n (%)	18,557 (13.5)	252 (17.0)	1285 (15.4)	3895 (19.0)	1231 (24.8)	396 (30.6)	229 (23.7)	< 0.001
GpIIb/IIIa-inhibitor, n (%)	34,771 (25.3)	318 (21.5)	1778 (21.3)	3889 (19.0)	707 (14.3)	196 (15.1)	123 (12.8)	< 0.001
Ventilation	23,872 (17.3)	312 (21.1)	1646 (19.7)	4913 (24.0)	1564 (31.5)	553 (42.7)	346 (35.9)	< 0.001
Stroke, n (%)	1917 (1.4)	33 (2.2)	166 (2.0)	534 (2.6)	149 (3.0)	53 (4.1)	19 (2.0)	< 0.001
In-hospital resuscitation, n (%)	13,940 (10.1)	173 (11.7)	823 (9.9)	2521 (12.3)	741 (14.9)	256 (19.8)	223 (23.1)	< 0.001
Blood transfusion, n (%)	11,327 (8.2)	193 (13.0)	1024 (12.3)	3364 (16.4)	1198 (24.2)	508 (39.2)	253 (26.2)	< 0.001
Bleeding, n (%)	9701 (7.0)	107 (7.2)	687 (8.2)	1926 (9.4)	521 (10.5)	189 (14.6)	89 (9.2)	< 0.001
Blood transfusion and/or bleeding, n (%)	18,124 (13.2)	260 (17.6)	1454 (17.4)	4412 (21.6)	1405 (28.3)	556 (42.9)	282 (29.2)	< 0.001
Sepsis, n (%)	2838 (2.1)	46 (3.1)	248 (3.0)	746 (3.6)	298 (6.0)	140 (10.8)	61 (6.3)	< 0.001
Renal replacement therapy, n (%)	2943 (2.1)	57 (3.9)	266 (3.2)	1032 (5.0)	567 (11.4)	674 (52.1)	575 (59.6)	< 0.001
Acute renal failure, n (%)	6808 (4.9)	139 (9.4)	729 (8.7)	2971 (14.5)	1,246 (25.1)	392 (30.2)	55 (5.7)	< 0.001
Acute renal failure and/or renal replacement therapy, n (%)	7,379 (5.4)	151 (10.2)	795 (9.5)	3,201 (15.6)	1,398 (28.2)	783 (60.5)	586 (60.7)	< 0.001
In-hospital death, n (%)	18,346 (13.3)	272 (18.4)	1,346 (16.1)	4,580 (22.4)	1,780 (35.9)	482 (37.2)	360 (37.3)	< 0.001
30-day mortality, n (%)	18,813 (13.7)	280 (18.9)	1,414 (16.9)	4,735 (23.1)	1,817 (36.6)	437 (33.8)	359 (37.2)	< 0.001

CKD denotes chronic kidney disease; Gp, glycoprotein, SD, standard deviation. Differences between CKD stages for categorical and continuous variables were tested via Chi-square test and Kruskal–Wallis test, respectively

### 30-day mortality

**Fig. 1:**
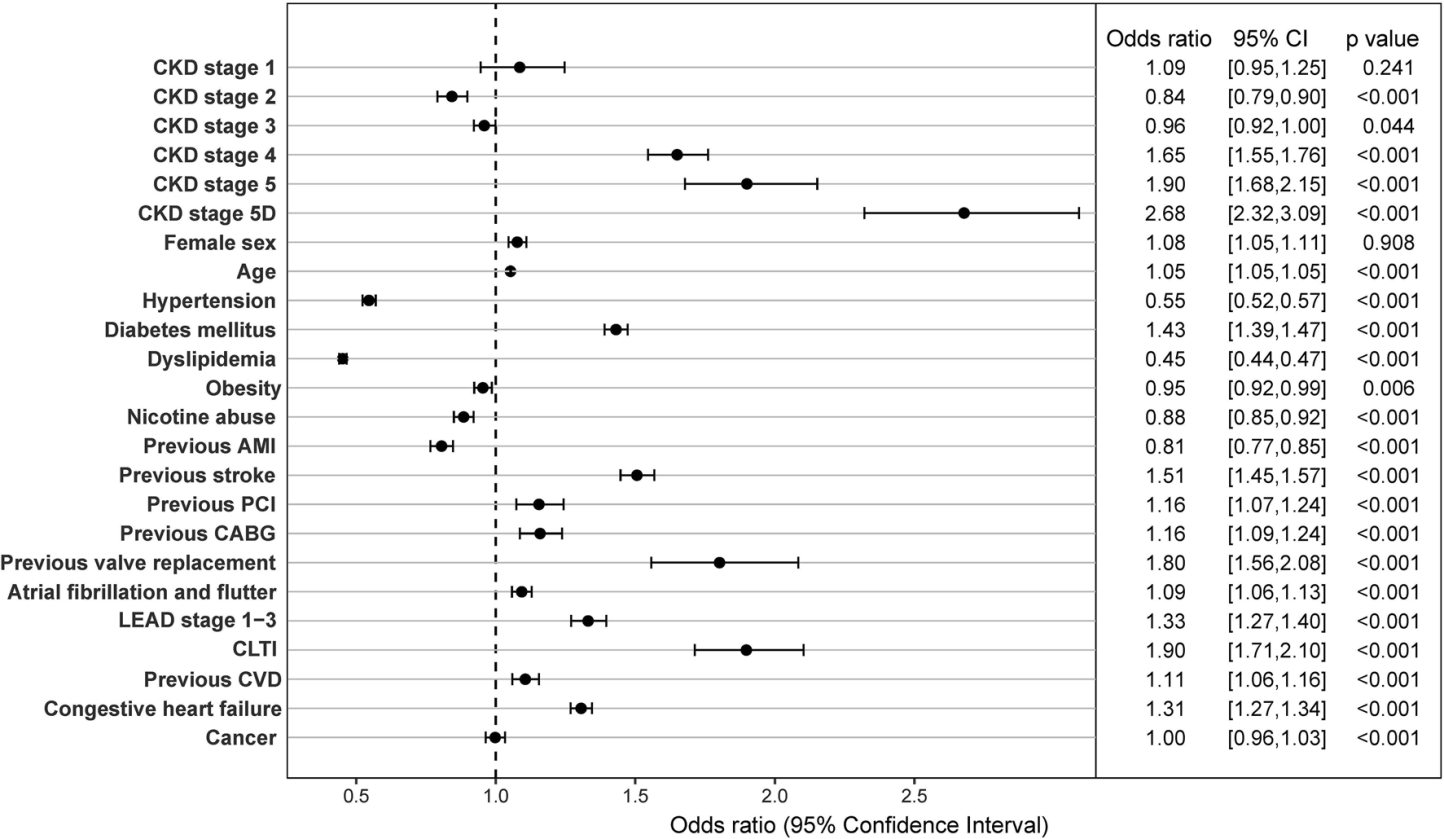
Risk factors for 30-day mortality. Multivariable logistic regression analysis of patients with ST-elevation myocardial infarction was performed to evaluate the association of chronic kidney disease (CKD) with 30-day mortality adjusted for age, sex, CKD stage at index and all the co-morbidities which are listed in the figure. Odd ratios are displayed with 95% confidence intervals (CI). AMI denotes acute myocardial infarction; CABG, coronary artery bypass grafting; CKD, chronic kidney disease; CLTI, chronic limb threatening ischemia; CVD, cerebrovascular disease; LEAD, low extremity artery disease; PCI, percutaneous coronary intervention

As shown in Table 2, 30 day-mortality was highest in patients with CKD stage 4, stage 5 and stage 5d (33.8%-37.2%) and lowest in patients without CKD (13.7%). Similarly, in the multivariable logistic regression analysis for 30-day mortality, CKD stage 4 (OR 1.65; 95% confidence interval (CI) 1.55–1.76), CKD stage 5 (OR 1.90; 95% CI 1.68–2.15), and CKD stage 5d (OR 2.68; 95% CI 2.32–3.09) as well as chronic limb-threatening ischemia (CLTI; OR 1.90; 95% CI 1.71–2.10), and previous valve replacement (OR 1.80; 95% CI 1.56–2.08, all *p* < 0.001) were the most prominent risk factors. Hypertension (OR 0.55; 95% CI 0.52–0.57) and dyslipidemia (0.45; 95% CI 0.44–0.47, both *p* < 0.001) were associated with a considerable risk reduction for 30-day mortality (Fig. 1).

### Medication after 1 year

One year after discharge, prescription rates for all four types of drug classes had markedly increased for all patient groups compared to medical treatment on admission (Table 3). Specifically, platelet inhibitors and beta-blockers were prescribed to at least 89% of all patients regardless of CKD status. Nevertheless, the trend to guideline-recommended medication preferably prescribed to patients with CKD had reversed, i.e., patients with the most severe kidney disease were least likely to receive all four drug classes (CKD stage 5: 59.4%, 95% CI 55.9–62.7; CKD stage 5d: 53.7%, 95% CI 49.7–57.6), whereas the percentage in patients without kidney disease rose up to 83.4% (95% CI 83.2–83.7; *p* < 0.001).

**Table 3:** Medication after one year

	No CKD	CKD Stage 1	CKD Stage 2	CKD Stage 3	CKD Stage 4	CKD Stage 5	CKD Stage 5D	*p* value
Oral anticoagulant, % (95%CI)	12.5 (12.3–12.7)	18.4 (16.3–20.6)	21.2 (20.2–22.1)	25.7 (25.0–26.3)	25.0 (23.5–26.5)	21.7 (18.9–24.6)	17.7 (14.8–20.8)	< 0.001
Platelet inhibitor, % (95%CI)	95.9 (95.8–96.0)	94.4 (93.6–94.7)	94.2 (93.6–94.7)	91.8 (91.4–92.3)	88.6 (87.5–89.7)	90.5 (88.3–92.3)	94.9 (92.8–96.4)	< 0.001
Oral anticoagulant and/or platelet inhibitor, % (95%CI)	96.6 (96.5–96.7)	95.1 (94.7–95.7)	95.2 (94.7–95.7)	92.7 (92.3–93.1)	88.7 (87.6–89.8)	89.6 (87.3–91.5)	92.1 (89.6–94.0)	< 0.001
Statin, % (95%CI)	93.7 (93.5–93.8)	90.0 (88.2–91.6)	90.7 (90.0–91.3)	85.1 (84.6–85.7)	78.2 (76.7–79.6)	77.6 (74.5–80.3)	75.2 (71.6–78.4)	< 0.001
Beta-blocker, % (95%CI)	93.2 (93.1–93.4)	93.3 (91.7–94.6)	93.6 (93.0–94.1)	92.3 (91.9–92.7)	90.4 (89.3–91.3)	90.1 (87.9–92.0)	90.9 (88.4–92.9)	< 0.001
ACE inhibitor or ARB, % (95%CI)	93.1 (93.0–93.3)	94.3 (92.8–95.5)	94.1 (93.6–94.7)	93.0 (92.6–93.4)	89.6 (88.5–90.6)	82.2 (79.4–84.7)	76.7 (73.1–79.9)	< 0.001
All four drug groups*, % (95%CI)	83.4 (83.2–83.7)	79.3 (76.9–81.5)	81.5 (80.6–82.4)	74.7 (74.0–75.4)	65.0 (63.3–66.7)	59.4 (55.9–62.7)	53.7 (49.7–57.6)	< 0.001

*The four drug groups are composed of (1) oral anticoagulant/platelet inhibitor, (2) statin, (3) beta blocker, (4) ACE inhibitor/ARB

ACE denotes angiotensin-converting-enzyme; ARB, angiotensin II receptor blocker; CKD, chronic kidney disease

Cumulative incidence functions to address the rates for medication after discharge determined by Nelson-Aalen estimates, where death was considered as a competing risk event. The presented rates represent the estimated rates of patients with at least one prescription in a one-year period after discharge. Differences between CKD stages were tested via Gray’s test

### Overall survival and MACE

**Fig. 2:**
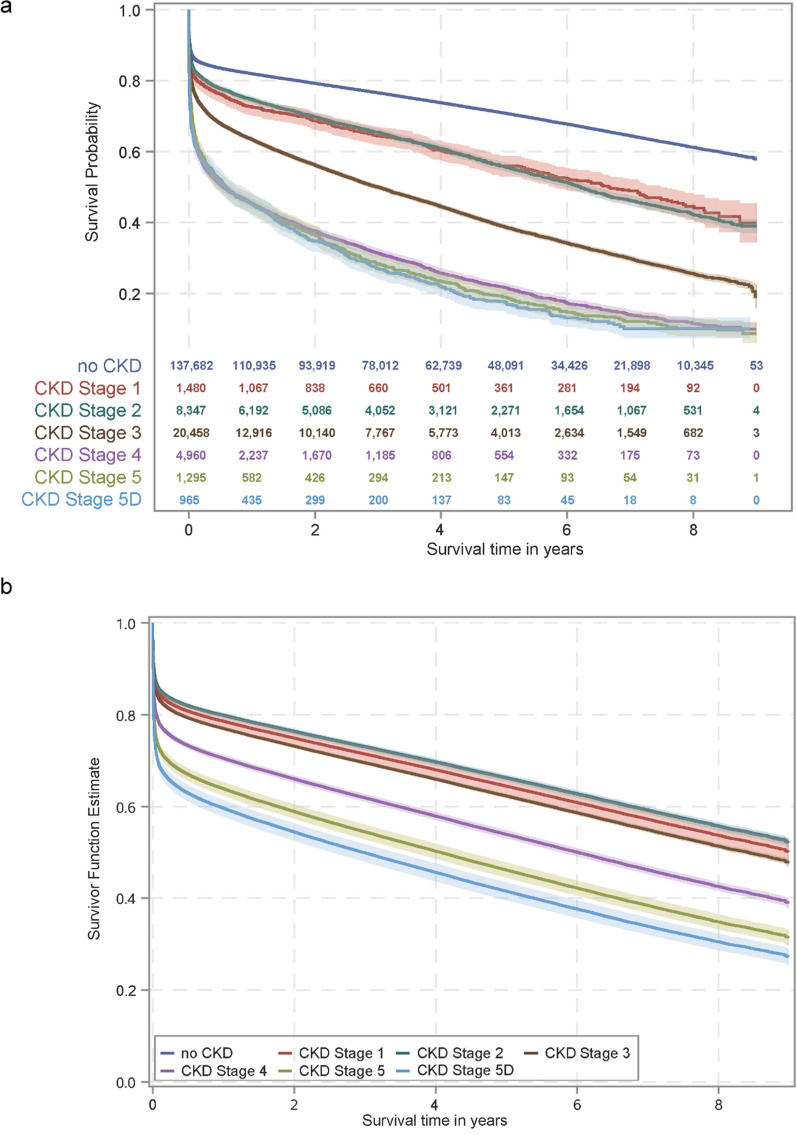
Survival of STEMI patients according to renal function. **a** Kaplan–Meier models of STEMI patients according to their renal function at index. Within nine years follow-up, the Kaplan–Meier survival estimates decreased from 58% without known CKD (no CKD) to 10% in STEMI patients on dialysis (CKD stage 5d). **b** Adjusted survival plot determined by Cox regression analysis for STEMI patients according to their renal function. Age, sex, CKD stage at index, and the patient's risk profile (hypertension, dyslipidemia, obesity, nicotine abuse, diabetes mellitus, chronic kidney disease, atrial fibrillation and/or flutter, peripheral artery disease, chronic heart failure, cerebrovascular disease, cancer, previous acute myocardial infarction, previous stroke, previous percutaneous intervention, previous coronary artery bypass grafting, previous valve replacement) were included in the model. The adjusted survival markedly decreased from 52% for patients without CKD (no CKD) and with CKD stage 2 (both curves overlap) to 27% for patients with dialysis-dependent CKD (CKD stage 5d). CKD denotes chronic kidney disease; STEMI, ST-elevation myocardial infarction

Patients were followed-up until 31 Dec 2018 corresponding to a minimal follow-up period of one year and a maximum follow-up period of nine years. Regarding overall survival, Kaplan–Meier survival estimates decreased with decreasing renal function (*p* < 0.001). While 58% of patients without CKD survived STEMI up to nine years after the index, the amount decreased to 45% and 39% for patients with CKD stage 1 and CKD stage 2, respectively, 19% for patients with CKD stage 3 and 11%, 9% and 10% for patients CKD stages 4, 5 and 5d, respectively (Fig. 2a). Adjusted survival plots yielded comparable results (Fig. 2b).

**Fig. 3:**
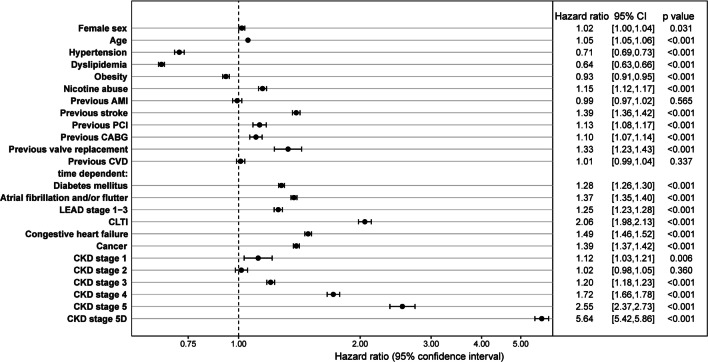
Time-dependent multivariable Cox regression for overall survival. Time-dependent multivariable Cox regression model was applied to evaluate the association of CKD stage and overall survival adjusted by patient risk profile. Worsening in the course of disease during follow-up is considered by time-dependent co-variables as indicated in the plot. All variable which are listed in the figure were included in the model. Hazard ratio, 95% confidence intervals (CI) and *p* values of risk factors of overall survival are displayed. AMI denotes acute myocardial infarction; CABG, coronary artery bypass grafting; CKD, chronic kidney disease; CLTI, chronic limb threatening ischemia; CVD, cerebrovascular disease; LEAD, low extremity artery disease; PCI, percutaneous coronary intervention

Multivariable time-dependent Cox regression analysis for overall survival identified the CKD stages 4, 5 and 5d as well as CLTI as the strongest independent predictors for poor overall survival (CKD stage 4: HR 1.72; 95% CI 1.66–1.78; CKD stage 5: HR 2.55; 95% CI 2.37–2.73; CKD stage 5d: HR 5.64; 95% CI 5.42–5.86; CLTI: 2.06; 95% CI 1.98–2.13; all *p* < 0.001, Fig. 3). Dyslipidemia (HR 0.64; 95% CI 0.63–0.66), arterial hypertension (HR 0.71; 95% CI 0.69–0.73), and obesity (HR 0.93; 95% CI 0.91–0.95, all *p* < 0.001) were associated with a protective effect on survival. No influence on overall survival could be noted for CKD stage 2, previous cerebrovascular disease and previous AMI (all *p* > 0.05). All other variables, e.g. older age, female sex, diabetes mellitus, and atrial fibrillation or flutter were associated with a decreased overall survival.

When analyzing the composite endpoint MACE, all CKD stages were associated with an adverse outcome (see Additional file 1: Fig. S1).

## Discussion

Based on a large, unselected cohort of STEMI patients, this study aimed to analyze the 30-day mortality and overall survival of STEMI patients with contemporary treatment in a high-income country, such as Germany. We found that CKD was a common concomitant disease and was associated with less guideline-recommended reperfusion therapies, more intensive care treatments, insufficient prescription of guideline-recommended medication during follow-up and a markedly increased mortality compared to no CKD. With decreasing renal function, the cumulative survival decreased from 60% in patients without CKD to 10% in patients with CKD stage 5d during the follow-up period of up to nine years. Especially the CKD stages 4, 5 and 5d as well as CLTI were independent predictors for 30-day mortality and overall survival.

Although randomized controlled trials are considered the gold standard for evaluating health care outcomes, the analysis of real-world data is also valuable to increase our knowledge of health care outcomes especially for patients who are usually excluded from randomized controlled studies. To the best of our knowledge, this real world analysis of 175,187 patients belongs to the largest cohorts of STEMI patients analyzed so far. The international GRACE registry enrolled more than 100,000 patients, about one third with STEMI, but the publications reported on sub-cohorts of 10,000 to 55,000 patients [9, 10]. The ongoing SWEDEHEART registry enrolls all hospitalized cases with acute coronary syndrome (up to 37,000 per year) in Sweden and analyses are performed on subgroups [14, 15]. Only the NCDR Chest Pain MI Registry reported on more included patients, namely 325,396 patients with STEMI, but CKD stages were not analyzed separately but on the basis of three groups consisting of patients either with preserved renal function, or moderate to severe renal function or on dialysis. [5]

In our analysis, more than 20% of all patients hospitalized with STEMI presented with concomitant CKD, thereof about 15% with CKD stages 3 or higher. Compared to other studies and registries, who reported proportions of 18% to 35% of STEMI patients with CKD stages 3 or higher [5, 10, 16, 17], the percentage is rather low. These differences might result from the different ways of data collection and cohort composition, e.g. including only patients with PCI as well as the difference in medical care in various countries. In our study, all patients in Germany of this distinct health insurance were included without prior selection. Despite these large differences in the prevalence of CKD that was reported in the studies mentioned above, CKD consistently is a frequent comorbidity in STEMI patients.

The high cardiovascular burden, the high frequency of in-hospital complications and the under-treatment of STEMI patients which has been known for several decades has been a matter of debate ever since [5, 10, 14, 18–20]. But there is still uncertainty to what extent the different CKD stages affect the prognosis. Most studies and registries of real-world data have differentiated only between patients without CKD, defined as an eGFR ≥ 60 ml/min, and with CKD, defined as eGFR < 60 ml/min [18], divided the cohort in three groups with eGFR ≥ 60 ml/min, eGFR 60–30 ml/min and eGFR < 30 ml/min [5, 10, 19], or focused only on dialysis-dependent patients [8, 9]. Seldomly, the five different stages were evaluated separately [14, 15, 21]. Furthermore, mostly one-year follow-up data are presented [12, 18, 22], longer follow-up periods are rare. [15, 19], Therefore, our analysis adds valuable data on 30-day mortality and overall survival up to nine years of STEMI patients according to their renal status. We could demonstrate that with increasing CKD stages the 30-day mortality rose up to 37.2% in dialysis-dependent patients and that up to nine years after STEMI, only about 10% of patients with CKD stages 4, 5 or 5d were still alive. The myocardial infarction was likely to be more severe in patients with higher CKD stages, as indicated by the higher percentages of, for example, cardiogenic shock, intra-aortic balloon pump, and ventilation in patients with more severe CKD. A former prospective Germany registry analyzed 4322 patients with acute myocardial infarction, but without cardiogenic shock, treated with primary PCI between 1998 and 2006 [11]. Of these, 39% suffered from STEMI. They also found an increasing mortality with decreasing renal function but 30-day mortality rates in patients with estimated creatinine clearance < 56 ml/min was only 7.7% [11], which is about half of the 30-day mortality rate of patients without CKD in our analysis. This might be due to the voluntary and not obligatory registry design and the exclusion of patients with cardiogenic shock. Even in the subgroup of patients older than 70 years, mortality rates were only 8.2%. Likewise, other studies and registries found lower in-hospital mortality rates: the large NCDR Chest Pain MI Registry reported in-hospital mortality rates between 2.4% in STEMI patients with eGFR ≥ 60 ml/min/1.73 m^2^ and 22.6% in dialysis-dependent patients [5]. In a Malaysian PCI registry, 13.2% of the patients with CKD stage 3 and higher died during hospital stay after STEMI [18]. In contrast to our analysis, the mentioned studies and registries included only patients who were treated with PCI. This could explain the dramatically increased 30-day mortality in our analysis. The findings of the Bremen STEMI registry support this assumption showing that a successful PCI was associated with improved in-hospital mortality. [12]

Whereas data on one-year mortality are frequent, data on overall- or long-term mortality covering a period longer than 5 years are rare, but their reported results are in accordance with our findings. The SWEDEHEART registry found mortality rates of 51.6% for women and 46.2% for men with CKD stages 3 and higher at a median follow-up period of about 3 years. [15] A Danish study presented 5-year all-cause mortality rates of 44.8% for STEMI patients with CKD stage 3 and 71.3% for STEMI patient with CKD stages 4 and 5, all treated with primary PCI [19]. Furthermore, Sabroe et al. found CKD stages 3 and higher to be independent predictors of 1-year mortality [19]. Likewise, our study could confirm that CKD stages 4, 5 and 5d were strong independent predictors of mortality. In addition, we could identify CLTI, a very serious atherosclerotic disease, which is often undertreated and has an extremely poor prognosis especially in patients with CKD [23, 24], as a strong predictor for poor overall survival. Both variables were time-dependent in the Cox regression analysis, therefore accounting for the worsening of the disease in the follow-up period.

Interestingly, the co-diagnoses hypertension, dyslipidemia and obesity showed protective effects on the outcome. Several other studies on patients with atherosclerotic disease have reported similar results [25–27]. These paradoxical findings might be explained by the medical treatment of hypertension and dyslipidemia and the worse outcome of cachectic patients compared to obese patients.

Patients with CKD stage 2 had no elevated risk for mortality, whereas CKD stage 1 or CKD stage ≥ 3 were independent predictors of mortality. We can only speculate what might be the cause of this effect. First, by definition of CKD stages, all patients with CKD stage 1 had proteinuria, which is a strong independent risk predictor of all-cause mortality, especially in patients with eGFR ≥ 90 ml/min/1.73m^2^, [28] whereas patients with CKD stage 2 did not necessarily have proteinuria. Second, patients with CKD stage 2 received coronary angiographies and PCIs slightly more often than patients with other CKD stages. Moreover, they experienced some complications less often, e.g. shock, blood transfusion, ventilation and acute renal failure, than patients with CKD stages 1 or 3 and higher. Third, in patients with renal insufficiency, patients with CKD stage 2 received most frequently all four guideline-recommended drugs one year after STEMI. All these factors might have influenced overall survival.

Guideline-recommended medical treatment of atherosclerotic diseases involves platelet aggregation inhibitors, blood pressure lowering medication and statins [2, 3]. These treatment strategies are only insufficiently applied to patients with advanced CKD, although patients with non-dialysis dependent CKD benefit from these drugs [29]. Sadly, the withholding of both interventions and medical treatment has a long history and has been suggested to contribute markedly to the worse outcome of renal patients [30]: already, an analysis of the treatment of 130,099 patients aged 65 years and older with a diagnosis of acute myocardial infarction between April 1994 and July 1995 described less frequent reperfusion and medical therapies with worsening renal function [20]. In particular, there should be more focus on the importance of long-term medical treatment, given the lack of alternative treatment options as reported in the recently published ISCHEMIA-CKD trial [31, 32]. The trial, including patients with stable coronary disease, moderate or severe ischemia and an eGFR < 30 ml/min/1.73m^2^, sought to determine whether an invasive strategy in combination with medical therapy was beneficial compared with conservative therapy alone. No evidence could be found that an initial invasive strategy reduced the risk of death or myocardial infarction in the 2.2 year follow-up period. However, the risk for stroke or the combined endpoint death or initiation of dialysis was higher in the invasive strategy group than in the group with medical treatment only [31]. A subgroup analysis of the ISCHEMIA-CKD trial found an association of higher numbers of guideline-directed medical therapy goals (low-density lipoprotein cholesterol < 70 mg/dL systolic blood pressure < 140 mm Hg, statin therapy, antiplatelet therapy, no smoking) with lower incidence of death and myocardial infarction [32]. Therefore, the knowledge gained during the last decades on drug treatment strategies for STEMI patients with CKD must be urgently implemented in daily practice. Cardiologists and nephrologists have different treatment foci due to their specialty which might have an influence on prescription pattern [33]. Whether the specialty of the treating physician has an impact on adherence is unclear, but there are many known factors associated with adherence, for example age, gender, socio-economic status, number of visits at the treating physician, complexity of dosing regimen, patient education, and co-morbidities. [34]

### Limitations

Our study has several limitations. First, analysis of diagnostic and procedure-based data always depends on the quality of the data. To ensure high-quality, we mainly focused on ICD and OPS codes which are unlikely to be incorrectly coded. These diagnostic and procedure codes are mandatory for complete and correct reimbursement of treatment costs. Particularly for the analysis of the outcome, we chose the endpoint “death” with an almost 100% probability of correct coding. Second, some variables as Killip classes or left ventricular ejection fraction which have been shown to be predictors of mortality in STEMI patients could not be analyzed due to the administrative nature of our data. Third, the retrospective design of our study has the same well-known disadvantages as every retrospective cohort study, e.g. associations, but no causations can be explored, and potential confounders e.g. socio-economic factors or different assessment and experience of the treating physician might generate bias.

## Conclusions

Our large real-data analysis of STEMI patients with and without CKD revealed an extremely high 30-day mortality and low overall survival for patients with advanced and dialysis-dependent CKD. Moreover, these patients are still undersupplied with revascularization therapies and drug treatment in contrast to all current guideline recommendations.

## Supplementary Information

**Additional file 1**: Supplementary Data. **Table S1:** ICD-10-GM and OPS codes. **Table S2**: Patients according to degree of chronic kidney disease and year of STEMI diagnosis. **Table S3**: Medication at admission. **Figure S1**: Time-dependent multivariable Cox regression for MACE.

## Data Availability

The authors confirm that the data utilized in this study cannot be made available in the manuscript, the supplemental files, or in a public repository due to German data protection laws (‘Bundesdatenschutzgesetz’, BDSG). Therefore, they are stored on a secure drive in the AOK Research Institute (WIdO), to facilitate replication of the results. Generally, access to data of statutory health insurance funds for research purposes is possible only under the conditions defined in German Social Law (SGB V § 287). Requests for data access can be sent as a formal proposal specifying the recipient and purpose of the data transfer to the appropriate data protection agency. Access to the data used in this study can only be provided to external parties under the conditions of the cooperation contract of this research project and after written approval by the sickness fund. For assistance in obtaining access to the data, please contact wido@wido.bv.aok.de.

## Abbreviations

ACE: Angiotensin-converting- enzyme
AOK: Allgemeine Ortskrankenkasse
ARB: Angiotension II receptor blocker
ATC: Anatomical Therapeutic Chemical
CI: Confidence interval
CKD: Chronic kidney disease
CLTI: Chronic limb-threatening ischemia
eGFR: Estimated glomerular filtration rate
G-DRG: German Diagnosis Related Groups
HR: Hazard ratio
IQR: Interquartile range
ICD-10 GM: International Statistical Classification of Diseases, German Modification
OPS: Operationen- und Prozeduren-Schlüssel
OR: Odds ratio
PCI: Percutaneous coronary intervention
STEMI: ST-elevation myocardial infarction
