# Supplementary material for: Contemporary in-hospital and long-term prognosis of patients with acute ST-elevation myocardial infarction depending on renal function: a retrospective analysis
Source: BMC Cardiovasc Disord. 2023 Feb 2;23:62. doi: 10.1186/s12872-023-03084-3 (PMC9896822; doi:10.1186/s12872-023-03084-3)
Supplement: Supplementary file 1 — Additional file 1: Supplementary Data. Table S1: ICD-10-GM and OPS codes. Table S2: Patients according to degree of chronic kidney disease and year of STEMI diagnosis. Table S3: Medication at admission. Figure S1: Time-dependent multivariable Cox regression for MACE. [file 12872_2023_3084_MOESM1_ESM.pdf]

Supplementary Data

Table of Contents

Table S1: ICD-10-GM and OPS codes

Analyzed diagnoses according to the ICD-10-GM (International Statistical Classification of Diseases, German Modification) codes and procedures according to the OPS (Operationen- und Prozeduren-Schlüssel, German procedure classification) codes.

Table S2: Patients according to degree of chronic kidney disease and year of STEMI diagnosis

Analyzed number of patients with a diagnosis of ST-elevation myocardial infarction according to the degree of chronic kidney disease for the years 2010 to 2017

Table S3: Medication at admission

Prescribed medication at admission according to the degree of chronic kidney disease

Figure S1: Time-dependent multivariable Cox regression for MACE

Time-dependent multivariable Cox regression model was applied to evaluate the association of CKD stage and MACE adjusted by patient risk profile. Worsening in the course of disease during follow-up is considered by time-dependent co-variables as indicated in the plot. All variable which are listed in the figure were included in the model. Hazard ratio, 95% confidence intervals (CI) and p values of risk factors of MACE are displayed.

Table S1: ICD-10-GM and OPS codes

Diagnosis	ICD-10-GM* or OPS code#
**Main diagnosis**	**ICD-10-GM code**
Acute ST-segment elevation myocardial infarction	I21.0, I21.1; I21.2, I22.0, I22.1, I22.8
**Secondary diagnoses**	**ICD-10-GM code**
Acute renal failure	N17*
Atrial fibrillation or atrial flutter	I48*
Bleeding	K92*, H44.8, T81.0, T81.2, T81.3, T81.7
Cancer	C*
Cerebrovascular disease	I65*, I66*, I67*
Chronic heart failure	I50*
Chronic kidney disease	
Stage 1	before 2010: N18.81
	since 2010: N18.1
Stage 2	before 2010: N18.82
	since 2010 N18.2
Stage 3	before 2010: N18.83
	since 2010 N18.3
Stage 4	before 2010: N18.84
	since 2010 N18.4
Stage 5	before 2010: N18.0
	since 2010 N18.5
	no OPS codes for dialysis
Stage 5d (dialysis-dependent)	8-853*, 8-854*, 8-855*, 8-857*
Coronary artery disease	I25*
One-vessel disease	I25.11
Two-vessel disease	I25.12
Three-vessel disease	I25.13
Diabetes mellitus	E10*, E11*, E12*, E13*, E14*
Dyslipidemia	E78*

Heart failure, left	
NYHA 1	I50.11
NYHA 2	I50.12
NYHA 3	I50.13
NYHA 4	I50.14
None	I50.19
Heart failure, right	I50.0*
Hypertension	I10*, I11*, I12*, I13*, I14*, I15*
Lower extremity artery disease (LEAD)	
LEAD 1-3 (mild LEAD)	before 2015:I70.20, I70.21
	since 2015: I70.20, I70.21, I70.22
LEAD 4-6 (severe LEAD or chronic limb	before 2015: I70.22, I70.23, I70.24
threatening ischemia (CLTI))	since 2015: I70.23, I70.24, I70.25
Myocardial infarction	I21*, I22*
Nicotine abuse	F17*
Obesity	E66*
Sepsis	A41*
Shock	R57.0, T81.1
Stroke	I60*, I61*, I62*; I63*, I64*, I69*
Hemorrhagic stroke	I60*, I61*, I62*
Ischemic stroke	I63*, I64
Valve replacement	Z95.2

**Procedures**	**OPS code**
Bare-metal stent	8-837.k*
Blood transfusion	8-800.c*
Coronary angiography	1-275.0*
Coronary artery bypass grafting	5-36*
Drug-eluting stent	8-837.m*
Extracorporeal membrane oxygenation	8-851*, 8-852*
GpIIb/IIIa-inhibitor	6-002.j*, 6.002.k*, 6.002.m*, 6.002.n*

Impella	8-83a.3*
In-hospital resuscitation	8-771
Intra-aortic balloon pump	8-83a.0*
Percutaneous coronary intervention	8-837*
Renal replacement therapy	8-853*, 8-854*, 8-855*, 8-857*
Thrombolysis	8-020.8

*ICD-10-GM codes start with a capital letter

[#]OPS-codes start with a number followed by a hyphen.

Table S2: Patients according to degree of chronic kidney disease and year of STEMI diagnosis

	No CKD	CKD Stage 1	CKD Stage 2	CKD Stage 3	CKD Stage 4	CKD Stage 5	CKD Stage 5D	All
Patients with STEMI diagnosis in 2010, n (%)*	18,717 (77.3)	250 (1.0)	1,379 (5.7)	2,777 (11.5)	711 (2.9)	271 (1.1)	99 (0.4)	24,204 (100.0)
Patients with STEMI diagnosis in 2011, n (%)*	18,432 (78.8)	209 (0.9)	1,147 (4.9)	2,642 (11.3)	655 (2.8)	176 (0.8)	136 (0.6)	23,397 (100.0)
Patients with STEMI diagnosis in 2012, n (%)*	18,248 (79.5)	144 (0.6)	998 (4.3)	2,532 (11.0)	691 (3.0)	192 (0.8)	140 (0.6)	22,945 (100.0)
Patients with STEMI diagnosis in 2013, n (%)*	17,716 (79.6)	105 (0.5)	877 (3.9)	2,642 (11.9)	632 (2.8)	148 (0.7)	126 (0.6)	22,246 (100.0)
Patients with STEMI diagnosis in 2014, n (%)*	17,241 (78.6)	169 (0.8)	978 (4.5)	2,653 (12.1)	615 (2.8)	131 (0.6)	128 (0.6)	21,915 (100.0)
Patients with STEMI diagnosis in 2015, n (%)*	16,392 (78.6)	190 (0.9)	1,012 (4.9)	2,443 (11.7)	575 (2.8)	130 (0.6)	117 (0.6)	20,859 (100.0)
Patients with STEMI diagnosis in 2016, n (%)*	15,795 (77.9)	181 (0.9)	1,023 (5.0)	2,487 (12.3)	576 (2.8)	120 (0.6)	102 (0.5)	20,284 (100.0)
Patients with STEMI diagnosis in 2017, n (%)*	15,141 (78.3)	232 (1.2)	933 (4.8)	2,283 (11.8)	505 (2.6)	126 (0.7)	117 (0.6)	19,337 (100.0)

* Percent values refer to the total number of patients of the corresponding year

CKD: chronic kidney disease; STEMI: ST-segment elevation myocardial infarction.

Table S3: Medication at admission

	No CKD	CKD Stage 1	CKD Stage 2	CKD Stage 3	CKD Stage 4	CKD Stage 5	CKD Stage 5D	p value
Patients, n (%)	137,682 (78.6)	1,480 (0.8)	8,347 (4.8)	20,458 (11.7)	4,960 (2.8)	1,295 (0.7)	965 (0.6)	
Previous oral anticoagulant, any, n (%)	4,906 (3.6)	107 (7.2)	717 (8.6)	2,281 (11.1)	577 (11.6)	119 (9.2)	97 (10.1)	<0.001
Previous platelet inhibitor, n (%)	17,162 (12.5)	323 (21.8)	1,929 (23.1)	5,465 (26.7)	1,447 (29.2)	444 (34.3)	547 (56.7)	<0.001
Previous oral anticoagulant, no platelet inhibitor, n (%)	4,212 (3.1)	95 (6.4)	585 (7.0)	1,851 (9.0)	484 (9.8)	84 (6.5)	56 (5.8)	<0.001
Previous platelet inhibitor, no oral anticoagulant, n (%)	16,468 (12.0)	311 (21.0)	1,797 (21.5)	5,035 (24.6)	1,354 (27.3)	409 (31.6)	506 (52.4)	<0.001
Previous oral anticoagulant and/or platelet inhibitor, n (%)	21,374 (15.5)	418 (28.2)	2,514 (30.1)	7,316 (35.8)	1,931 (38.9)	528 (40.8)	603 (62.5)	<0.001
Previous ACE inhibitor or ARB, n (%)	57,247 (41.6)	937 (63.3)	5,114 (61.3)	14,119 (69.0)	3,568 (71.9)	809 (62.5)	532 (55.1)	<0.001
Previous beta-blocker, n (%)	42,157 (30.6)	682 (46.1)	3,975 (47.6)	11,330 (55.4)	2,996 (60.4)	795 (61.4)	679 (70.4)	<0.001
Previous statin, n (%)	25,766 (18.7)	473 (32.0)	2,624 (31.4)	6,768 (33.1)	1,614 (32.5)	497 (38.4)	418 (43.3)	<0.001
Previous prescription of any of the four drug groups*, n (%)								<0.001
None	60,726 (44.1)	299 (20.2)	1,890 (22.6)	2,961 (14.5)	563 (11.4)	154 (11.9)	67 (6.9)	
1 drug group	34,011 (24.7)	412 (27.8)	2,061 (24.7)	4,870 (23.8)	1,121 (22.6)	288 (22.2)	178 (18.4	
2 drug groups	23,263 (16.9)	356 (24.1)	1,979 (23.7)	5,718 (27.9)	1,489 (30.0)	389 (30.0)	268 (27.8)	
3 drug groups	12,721 (9.2)	266 (18.0)	1,460 (17.5)	4,409 (21.6)	1,138 (22.9)	293 (22.6)	290 (30.1)	
4 drug groups	6,961 (5.1)	147 (9.9)	957 (11.5)	2,500 (12.2)	649 (13.1)	171 (13.2)	162 (16.8)	

*The four drug groups are composed of (1) oral anticoagulant/platelet inhibitor, (2) statin, (3) beta blocker, (4) ACE inhibitor/ARB.

ACE denotes angiotensin-converting-enzyme; ARB, angiotensin II receptor blocker; CKD, chronic kidney disease.

Figure S1: Time-dependent multivariable Cox regression for MACE

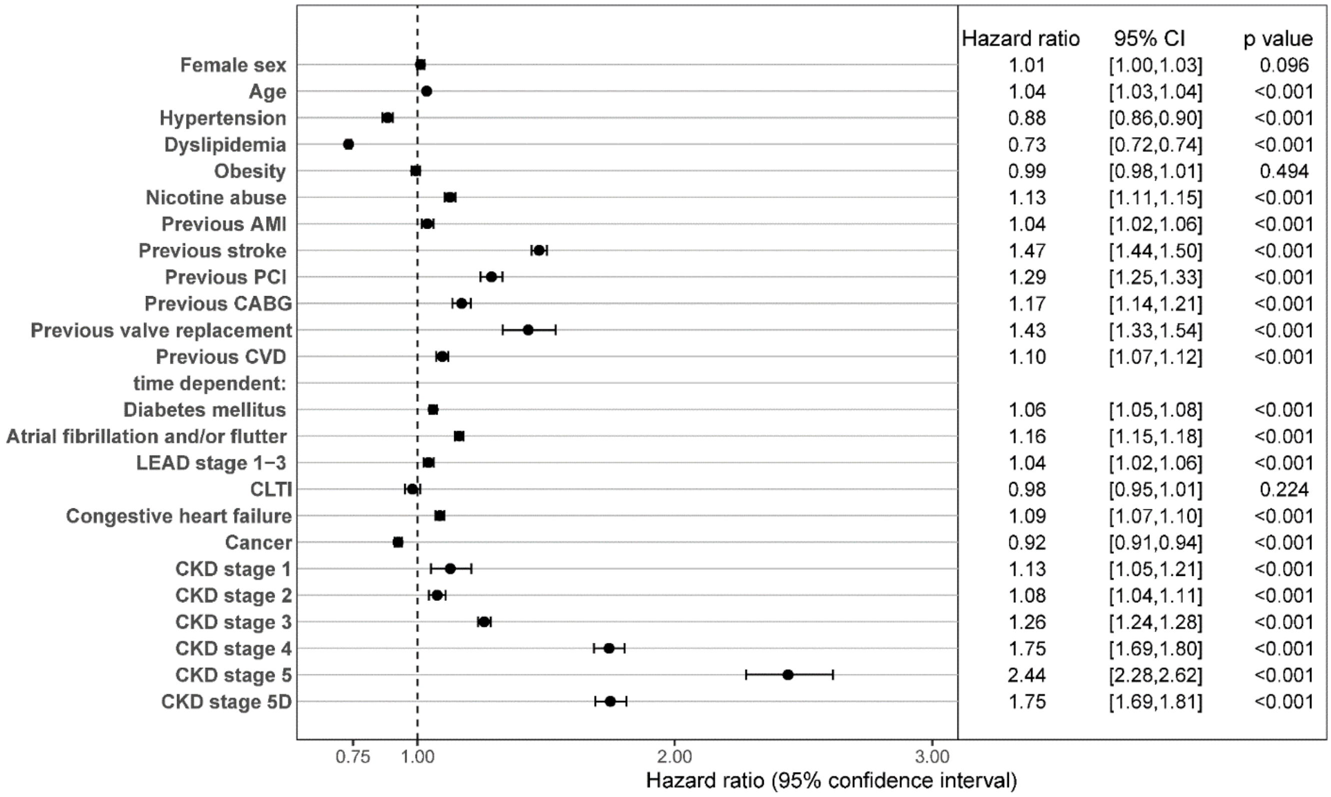

Time-dependent multivariable Cox regression model was applied to evaluate the association of CKD stage and MACE adjusted by patient risk profile. Worsening in the course of disease during follow-up was considered by time-dependent co-variables as indicated in the plot. All variable which are listed in the figure were included in the model. Hazard ratio, 95% confidence intervals (CI) and p values of risk factors of MACE are displayed.

AMI denotes acute myocardial infarction; CABG, coronary artery bypass grafting; CKD, chronic kidney disease; CLTI, chronic limb threatening ischemia; CVD, cerebrovascular disease; LEAD, low extremity artery disease; MACE, major adverse cardiovascular events (defined as myocardial infarction, stroke, resuscitation, death); PCI, percutaneous coronary intervention
